# *Encephalitozoon cuniculi*: Grading the Histological Lesions in Brain, Kidney, and Liver during Primoinfection Outbreak in Rabbits

**DOI:** 10.1155/2016/5768428

**Published:** 2016-02-28

**Authors:** Luis E. Rodríguez-Tovar, Alicia M. Nevárez-Garza, Armando Trejo-Chávez, Carlos A. Hernández-Martínez, Gustavo Hernández-Vidal, Juan J. Zarate-Ramos, Uziel Castillo-Velázquez

**Affiliations:** ^1^Cuerpo Académico de Zoonosis y Enfermedades Emergentes, Facultad de Medicina Veterinaria y Zootecnia, Universidad Autónoma de Nuevo León, Calle Francisco Villa s/n, Ex-Hacienda El Canadá, 66050 Escobedo, NL, Mexico; ^2^Cuerpo Académico de Nutrición y Forrajes, Facultad de Agronomía, Universidad Autónoma de Nuevo León, Calle Francisco Villa s/n, Ex-Hacienda El Canadá, 66050 Escobedo, NL, Mexico; ^3^Cuerpo Académico de Patobiología, Facultad de Medicina Veterinaria y Zootecnia, Universidad Autónoma de Nuevo León, Calle Francisco Villa s/n, Ex-Hacienda El Canadá, 66050 Escobedo, NL, Mexico; ^4^Cuerpo Académico de Epidemiología Veterinaria, Facultad de Medicina Veterinaria y Zootecnia, Universidad Autónoma de Nuevo León, Calle Francisco Villa s/n, Ex-Hacienda El Canadá, 66050 Escobedo, NL, Mexico

## Abstract

This is the first confirmed report of* Encephalitozoon cuniculi* (*E. cuniculi*) in farm meat rabbits located in Northern Mexico. Eighty young rabbits exhibited clinical signs of this zoonotic emerging disease, like torticollis, ataxia, paresis, circling, and rolling. Samples of brain, kidney, and liver were examined for histology lesions. For the first time the lesions caused by* E. cuniculi* were graded according to their severity (I, II, and III) and the size of the granulomas (Types A, B, and C). The main cerebral injuries were Grade III, coinciding with the presence of Type C granulomas. The cerebral lesions were located in the cortex, brain stem, and medulla. The renal lesions were also Grade III distributed throughout cortex and renal medulla, with no granuloma formation. The involvement of hypersensitivity Types III and IV is suggested. All of the rabbits were seropositive to* E. cuniculi* by CIA testing, suggesting that this zoonotic and emerging pathogen is widely distributed among animals intended for human consumption. We believe this work could be used as a guide when examining* E. cuniculi* and will provide direction to confirm the diagnosis of this pathogen.

## 1. Introduction

*Encephalitozoon cuniculi* (*E. cuniculi*) is a single-celled obligate intracellular microorganism belonging to the phylum Microsporidia, class Microsporea, and order Microsporidia [1, 2]. All mammals are susceptible to infection with this eukaryotic organism [3]; however, it causes significant central nervous system (CNS) and renal disease in pet and laboratory rabbits (*Oryctolagus cuniculus*) [4]. Infected rabbits eliminate the spores in urine and feces; thus, infection of the host generally occurs after the ingestion of water or food contaminated with infective spores [5].* Encephalitozoon cuniculi* is considered to be an emergent, zoonotic, and opportunistic pathogen in immunocompetent and immunocompromised individuals [6, 7]. The spores infect enterocytes and, perhaps through Peyer's patches or interepithelial lymphocytes, spread to the bloodstream or the lymphatic system, reaching the brain, kidney, liver, and other organs. Spores can also be internalized by intercellular phagocytes, which are located in the intestinal epithelium, and from there they migrate to other organs [8]. Intrauterine infection has also been suggested as an important route of transmission to offspring, which occasionally develop cataracts, uveitis, and hypopyon [9]; inhalation of microsporidian spores is another possible route of contagion [10].* Encephalitozoon cuniculi* infection causes a severe granulomatous meningoencephalitis and a chronic interstitial nephritis and fibrosis [11]. In many rabbits, the disease can persist subclinically for months. Due to brain damage, infected rabbits can develop signs of central vestibular disease, which is characterized by torticollis, ataxia, paresis, and longitudinal rolling; the animal is often unable to right itself and even be nystagmus and seizures [12]. Chronic renal failure, although quite common in infected rabbits, tends to result in less specific symptoms, such as weight loss, lethargy, and loss of appetite. Some rabbits may also develop urinary incontinence and can become polydipsic and polyuric [4]. Typical kidney lesions manifest as depressed scars on the outer surface, which are more evident when the capsule is detached. These are caused by a chronic interstitial nephritis and fibrosis, giving the kidney a pitted appearance [13].* Encephalitozoon cuniculi* has become an important opportunistic pathogen for immunosuppressed individuals, such as AIDS patients and those who receive antitumour or immunosuppressive drugs [14, 15]. Additionally, this microorganism can also infect immunocompetent persons and animals [16]. For these reasons,* E. cuniculi* has garnered increased attention worldwide and has emerged as an important pathogen for both humans and animals [17]. There have been a number of recent immunological and pathological studies in Europe, Asia, and US, which focused on* E. cuniculi* infections in rabbits and the people surrounding these animals, like works and researchers [18, 19]. Most of these studies involved rabbits, either as pets or those from laboratory animal centers [12]. However, no serological or histopathological studies of this zoonotic disease have been conducted in Mexico. Therefore, the purpose of this study was to fully characterize the histological lesions, along with the results of serological, coprological, and urine analysis from a severe outbreak of encephalitozoonosis in meat rabbit farms, where this pathogen has not previously been seen.

## 2. Materials and Methods

### 2.1. Animals for Study

Eighty New Zealand white rabbits from five semi-intensive farms, destined for meat purposes, were examined after presenting with neurological clinical signs suggestive of encephalitozoonosis, such as torticollis, lethargy, rolling, or walking in circles. Twenty-eight rabbits (13 females, 15 males) had indirect evidence of polyuria, characterized by dehydration and urine scalding of posterior limbs. The farms were established in the municipalities of Cadereyta Jiménez (25°36′N; 100°00′W), Ciénega de Flores (25°57′N; 100°11′W), Higueras (25°57′N; 100°01′W), Marín (25°23′N; 100° 02′W), and Galeana (24°50′N; 100°04′W), Nuevo León, México, and the infection occurred during June to August 2015. The animals were young, with an age of approximately 3 months, and both sexes were affected (35 females and 45 males). All of them presented with severe weight loss, approximately 1.5 kg (normal = 2.5–3 kg) and were supposedly ready for the meat market. Sick animals were submitted for postmortem examination to the Pathology Department of the Facultad de Medicina Veterinaria y Zootecnia, Universidad Autónoma de Nuevo León.

### 2.2. Collection of Samples and Histology

The rabbits were euthanized, and a routine postmortem examination was performed. During necropsy, the outer and inner ears and tympanic bulla were examined to discriminate a possible infection of the ear canals. Samples from the brain, kidney, lungs, heart, liver, digestive system, and eyes were fixed in 10% neutral buffered formalin for 48 hours. The samples were processed for routine histology and stained with hematoxylin and eosin (HE), calcofluor white (CW), and modified trichrome blue (MTB). A positive infection with* E. cuniculi* was considered when spores were observed in the organs examined.

*Central Nervous System (CNS).* Histological examination of the brain included the meninges, frontal lobe, cerebellum, thalamus, hypothalamus, pons, midbrain, medulla oblongata, brain stem, and the cervical region of spinal cord. To evaluate and characterize the lesions observed in the brain, we used the following: Grade I, generalized vascular congestion, perivascular and perineural oedema, and discrete lymphocytic cuffing; Grade II, moderate nonsuppurative encephalitis and glial reaction, astrocytes, astrogliosis and moderate perivascular lymphocytic infiltrate, and parasitic cysts; Grade III, severe nonsuppurative granulomatous meningoencephalitis with adjacent parasitic cysts, numerous areas of glial reaction, abundant perivascular lymphocytic infiltrate, satellitosis, hemorrhage, neuronal necrosis, and malacia. Similarly, the sizes of the granulomas were classified as follows: Type A, granulomas under 50 *μ*m in diameter; Type B, granulomas of 100 *μ*m in diameter; and Type C, granulomas greater than 100 *μ*m in diameter.

*Renal Tissue*. Similarly, lesions in kidneys were classified as follows: Grade I, interstitial nephritis, characterized by a discrete lymphocytic infiltration and degenerative changes in the epithelial cortex and medulla, dilation of renal glomeruli, and hyaline casts within the tubular lumen; Grade II, moderate chronic interstitial nephritis with necrosis and desquamation of the tubular epithelium; Grade III, severe chronic nonsuppurative granulomatous interstitial nephritis, accompanied by necrosis, glomerular synechiae, inter- and intratubular hemorrhage, and severe interstitial necrosis and fibrosis.

*Hepatic Tissue*. The same tissue grading system was used as follows: Grade I, nonsuppurative periportal interstitial infiltration with centrilobular congestion and telangiectasia; Grade II, the previous described lesions accompanied by fatty change, congestion, and centrilobular ballooning degeneration; Grade III, centrilobular necrosis and hemorrhage.

### 2.3. Carbon Immunoassay (CIA)

Blood samples were taken from the central auricular artery of all rabbits. Clot formation was allowed, and sera were stored at −20°C until the carbon immunoassay (CIA) test (Medicago®, Uppsala, Sweden) was performed. The assay is based on the detection of IgG specific for* E. cuniculi* spores and was performed according to the manufacturer's instructions. The results were read under a light microscope at 40x. Sera from rabbits with agglutinated spores that stained greyish-brown against a carbon particle background were considered positive for* E. cuniculi* infection. A negative result was considered when spores appeared whitish against the same background. All samples were compared to positive and negative sera controls from the commercial kit.

### 2.4. Collection of Urine and Stools

Both procedures were performed during necropsy. Urine samples were collected by cystocentesis and stored at 4°C prior to staining with calcofluor white and trichrome blue. Fecal samples were taken directly from the rabbit's intestine at necropsy and were placed directly into plastic containers containing 10% neutral buffered formalin. Samples were screened for the presence of* E. cuniculi* spores using the same staining procedures as utilized for the urine samples.

*CW Staining*. Methanol-fixed fecal smears were stained with calcofluor white for 10 min in a dark room and rinsed with distilled water. The slides were then air-dried and mounted with antifading aqueous mounting medium, containing DABCO® antifading (Sigma-Aldrich, México). Samples were observed under a UV epifluorescence microscope at a wavelength of 395 to 415 nm. In positive samples, spores appeared as bluish-white or turquoise oval structures against a dark background.

*MTB*. Methanol-fixed fecal smears were stained with trichrome blue (Para-Pak®, Meridian Bioscience, Inc., Cincinnati, OH) for 90 min at 37°C. Slides were then rinsed sequentially in acid alcohol, ethanol, and xylene, mounted in histological resin, and observed under a light microscope at 100x. Spore quantification was performed as previously described, and samples were given one of the four designations based on the number of spores observed: (a) many (more than 10 spores), moderate (5–10 spores), rare (about 1–5 spores), and indeterminate (no spores). In positive samples, spores are observed as bright pinkish-red ovals or rounded structures, sometimes displaying a pinkish-red belt-like stripe.

### 2.5. Statistical Analysis

A chi-squared analysis was used to test for the differences in infection rates among the different groups of rabbits and to identify any association between the lesion grade and rabbit gender. A level of *p* < 0.05 was considered significant. The software Minitab v.16 (Minitab® Inc., Pennsylvania, USA) was used for statistical analysis.

## 3. Results

### 3.1. Clinical Presentation

All of the rabbits examined in this study had neurological symptoms compatible with* E. cuniculi* infection. Varying degrees of central vestibular disorders were observed, including head tilt, circling, rolling, ataxia, nystagmus, and paresis. During postmortem examination, none of the rabbits displayed signs of head trauma, dental diseases, tympanic bulla problems, ear mites, or ear canal bacterial infection, any of which could have explained the neurological symptoms observed.

### 3.2. Carbon Immunoassay (CIA) Test

All sampled rabbit sera were positive for* E. cuniculi* by the CIA test. In all cases, numerous spores were observed under the 40x objective; spores were surrounded with carbon particles in a brownish or sepia background, which indicates the presence of IgG reactive against* E. cuniculi* spores.

### 3.3. Histopathological Findings

**Figure 1:**
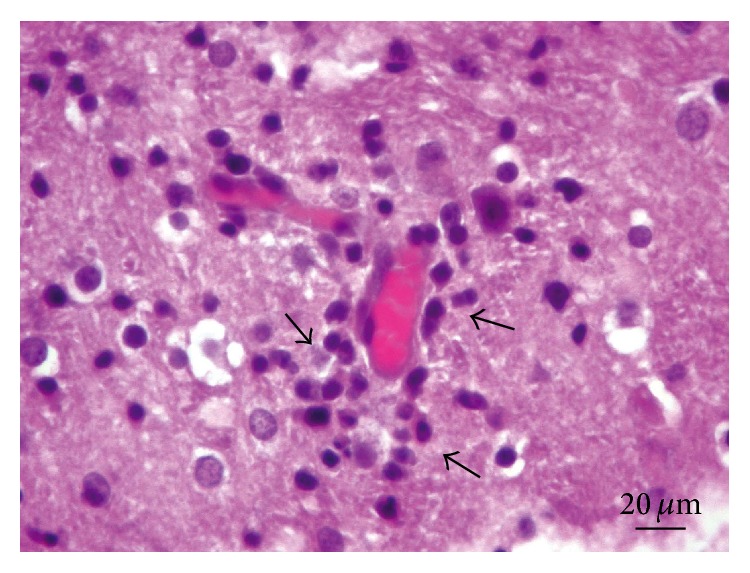
Brain. Lesion Grade I. Vascular congestion and discrete lymphocytic cuffing (arrows). Hematoxylin and eosin.

**Figure 2:**
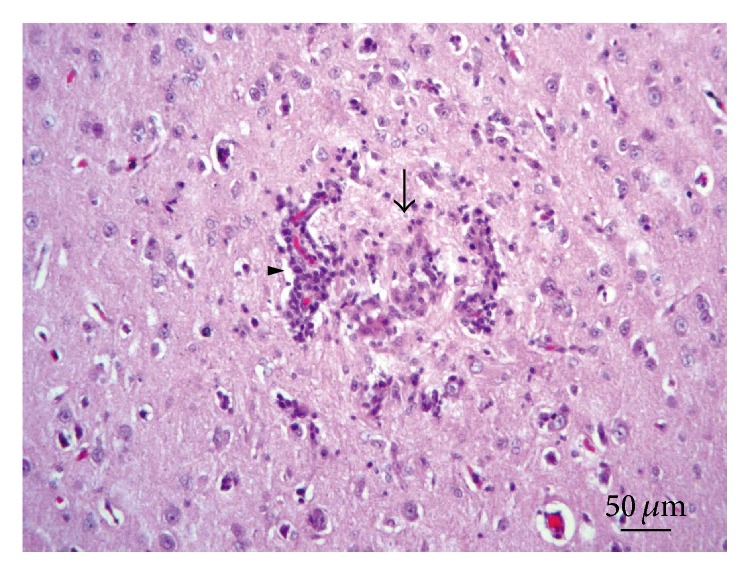
Brain. Lesion Grade II. Moderate granulomatous reaction (arrow) accompanied with glial reaction. Perivascular lymphocytic infiltrate (arrowhead). Hematoxylin and eosin.

**Figure 3:**
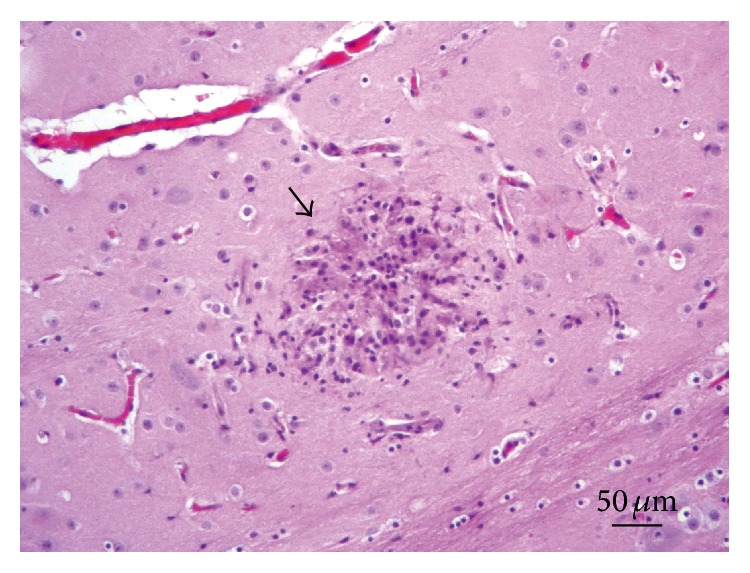
Brain. Lesion Grade III. Severe nonsuppurative granulomatous reaction (arrow) composed by microglial cells and mononuclear cells (lymphocytes). Hematoxylin and eosin.

*Central Nervous System.* All the examined rabbit brain samples showed microscopic evidence of chronic cerebral injury, characterized by multifocal nonsuppurative granulomatous encephalomyelitis, accompanied by* E. cuniculi* cysts. The most affected sites were the cerebral cortex, white matter, and medulla oblongata, while the cerebellum, spinal cord, and meninges did not show microscopic lesions or parasitic cysts in any of the examined specimens. Thirty rabbits (38%) had Grade I lesions (Figure 1), 16 animals (20%) showed Grade II lesions (Figure 2), and 35 rabbits (44%) had Grade III lesions (Figure 3). Any differences observed between rabbit groups and gender were not statistically significant (*χ* ^2^ = 5.64; *p* = 0.05). Further, adjacent to Type C granulomas, we observed both intact and ruptured parasitic cysts releasing infective spores directly into the nervous tissue (Table 1).

**Figure 4:**
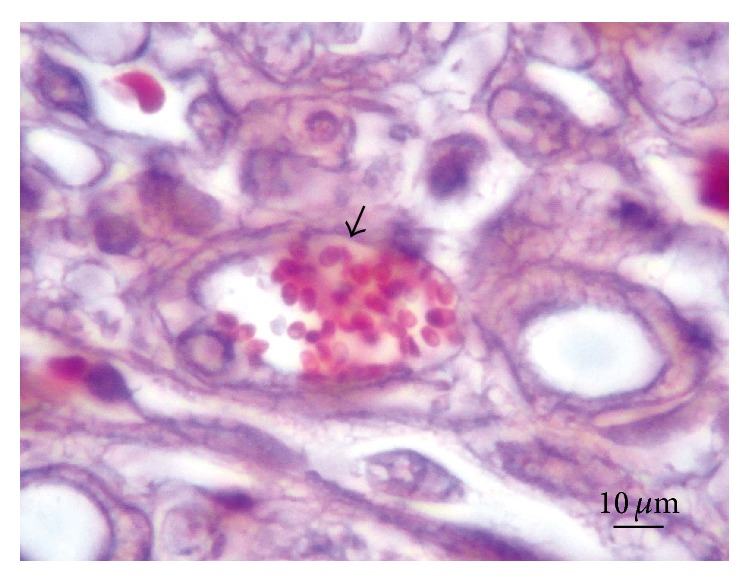
Kidney. Intraluminal* Encephalitozoon cuniculi* spores (arrow). Modified trichrome blue stain.

**Figure 5:**
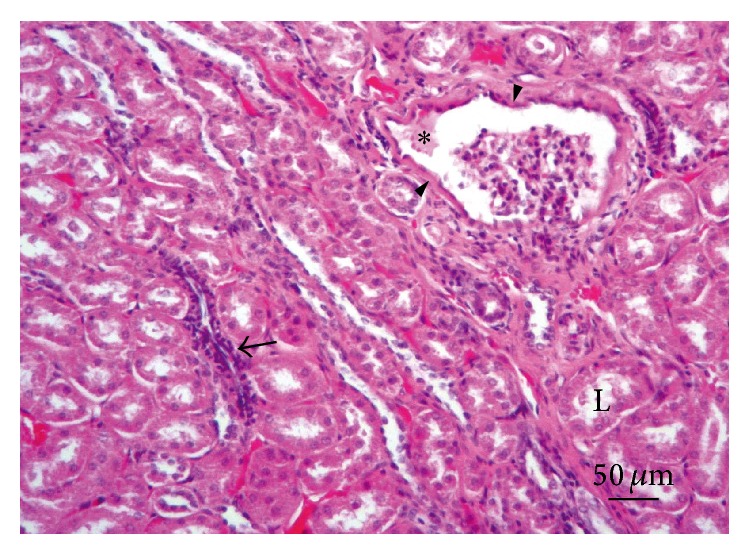
Kidney. Lesion Grade I. Mild interstitial mononuclear cells infiltration (arrow). Thickening of Bowman's capsule (arrowheads). Evidence of protein in Bowman's space (*∗*). Degenerative changes of renal tubules (L). Hematoxylin and eosin.

**Figure 6:**
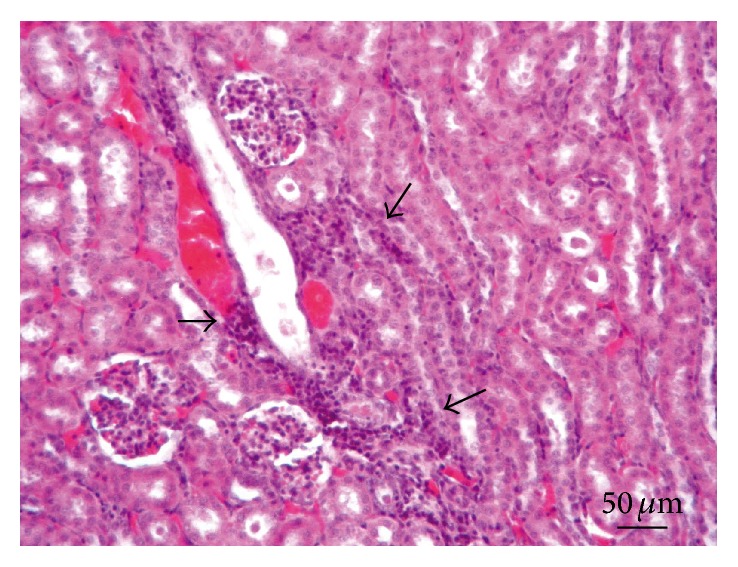
Kidney. Lesion Grade II. Abundant interstitial mononuclear cells infiltration (arrows) accompanied by tubules necrosis. Note the dilation of glomeruli occupying entirely Bowman's space. Hematoxylin and eosin.

**Figure 7:**
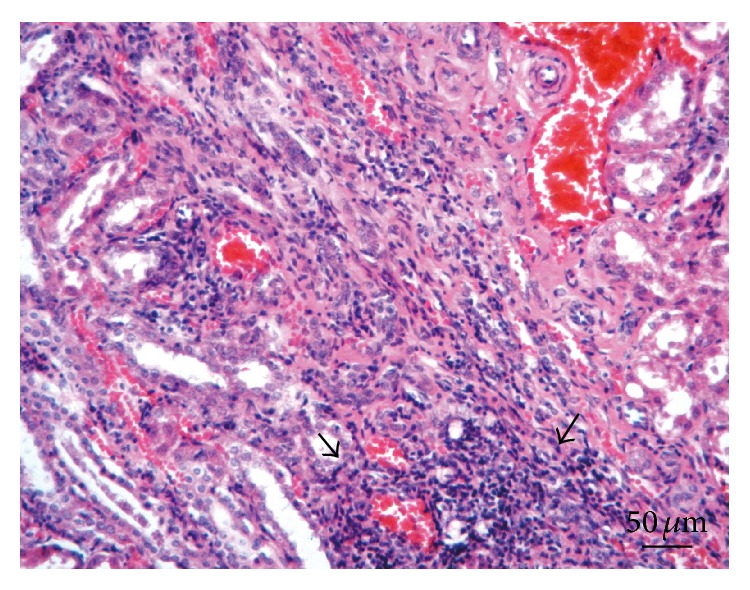
Kidney. Lesion Grade III. Severe interstitial mononuclear cells infiltration (arrows) with fibrosis, necrosis, atrophy, dilation, and degenerative changes of tubular epithelium. Congestive changes in the right upper part of the micrograph. Hematoxylin and eosin.

*Kidneys*. Macroscopically, renal tissue samples showed evidence of chronic renal lesions. In 35 animals (44%), there were multiple foci containing dark red and grey irregular pitted areas, approximately 0.5–1 cm, on the outer kidney surfaces. In severe cases, these injuries often combined to form larger lesions. The cutting surface of the renal cortex showed radial greyish-white areas extending into the medulla, while the renal pelvis displayed no visible changes. Microscopically, renal lesions were characterized by multifocal and segmental nonsuppurative granulomatous interstitial nephritis, associated with* E. cuniculi* cysts and free spores (Figure 4). In the renal parenchyma, thirty-two rabbits (40%) had Grade I lesions (Figure 5), 10 animals (13%) contained Grade II lesions (Figure 6), and 38 rabbits (48%) had Grade III lesions (Figure 7). The differences among groups and gender were not statistically significant (*χ* ^2^ = 9.52; *p* = 0.17). Additionally, parasitic cysts in the tubular epithelium containing abundant* E. cuniculi* spores were observed in rabbits with lesions characterized as Grades II or III.

**Figure 8:**
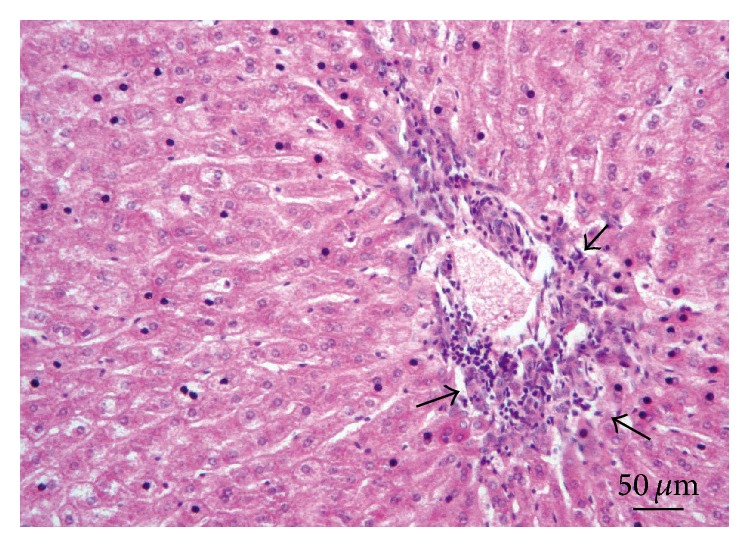
Liver. Lesion Grade I. Mild nonsuppurative periportal interstitial infiltration (arrow) and initial hepatocytes degenerative changes. Hematoxylin and eosin.

**Figure 9:**
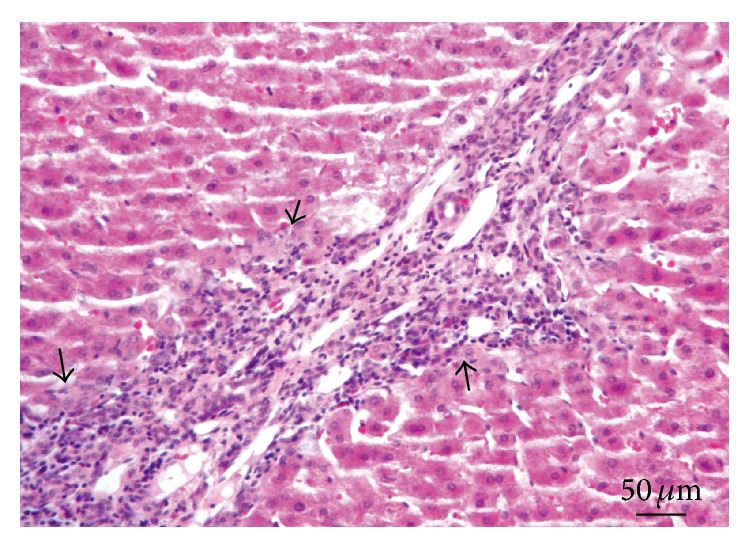
Liver. Lesion Grade II. Moderate nonsuppurative periportal interstitial inflammatory response (arrows). Hematoxylin and eosin.

**Figure 10:**
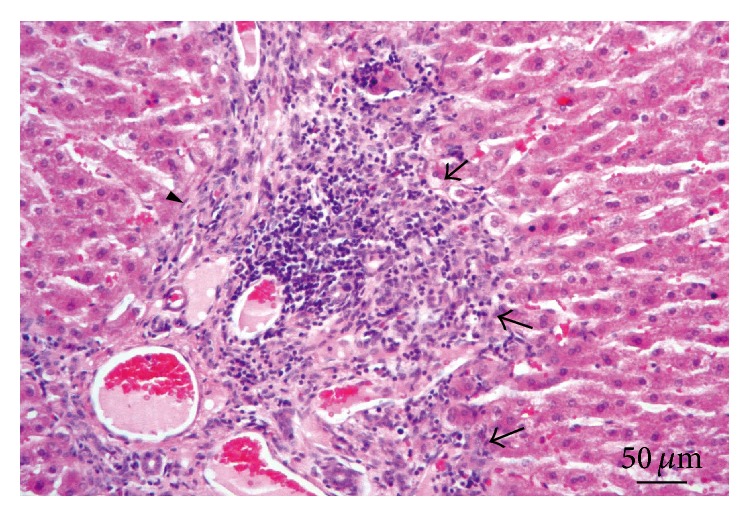
Liver. Lesion Grade III. Severe periportal nonsuppurative interstitial infiltration (arrows), accompanied by fibrosis and necrosis of periportal area (arrowhead). Hematoxylin and eosin.

*Liver*. The livers of all animals examined showed macroscopic and microscopic evidence of injury. At necropsy, the livers were congested and enlarged, and several of them were hardened. Glisson's capsule was thickened and opalescent. Hepatic lesions were characterized by a multifocal nonsuppurative granulomatous inflammatory response. Sixteen rabbits (20%) showed Grade I lesions (Figure 8), 37 animals (47%) had Grade II lesions (Figure 9) with hepatic fatty changes, and 27 rabbits (34%) had Grade III lesions (Figure 10) in the hepatic parenchyma. The differences among groups and gender were not statistically significant (*χ* ^2^ = 6.456; *p* = 0.06).

### 3.4. Urine Examination

*MTB*. We found that 23% of the rabbits tested positive for the presence of spores in the urinary sediment. The spores were scarce, with 2-3 per field, approximately 2–4 *μ*m in length, and were pink to reddish pink. Most were oval or slightly elongated.

*CW*. This fluorochrome enabled the rapid observation of microsporidian spores in 29% of rabbits. The spores were easily observed and stained as an intense bright blue turquoise against a dark background.

### 3.5. Stool Examination

*MTB*. All sampled rabbits were positive for microsporidian spores in the stool. These stained bright red to pink and were measured approximately 2–4 *μ*m in length. Numerous spores showed the typical diagonal or horizontal line in the equatorial belt, denoting the polar tube. We found that 59 rabbits (74%) showed 1 to 5 spores per field (rare), 12 animals (15%) showed 5 to 10 spores per field (moderate), and 9 animals (11%) had more than 10 spores per field (many), Table 2.

*CW.* The staining of fecal samples with this fluorochrome allowed the rapid observation of microsporidian spores in all rabbits. Due to the dark background of the smear, identification of spores was easy. Mature and complete spores showed an intense bright blue fluorescence, whereas incomplete or germinated spores had a faint bluish color.

## 4. Discussion

In this study, we assessed 80 rabbits from a severe outbreak of* E. cuniculi* in young rabbits. All our data strongly implicate this pathogen as the causative agent of disease, and the microorganism was identified by (1) the morphological features of the spores in the brain and the kidneys, using different stains [20]; (2) the histological injuries in the organs sampled [4]; (3) detection of spores in stools and urine using the MTB and CW staining techniques [21]; and (4) serological confirmation by CIA testing [22]. In this study, surprisingly all animals studied showed severe cerebral lesions compatible with* E. cuniculi*. The most affected sites were the brain stem, medulla oblongata, and cerebral cortex. Contrary with the literature, the cerebellum, meninges, and spinal cord remained without pathological changes. No injury was observed in the ear canals, tympanic bulla, or vestibular bodies, which is surprising in light of the fact that the rabbits displayed numerous signs of central vestibular disease often found in conjunction with cerebellar lesions, including ataxia, rolling and circling, lethargy, and torticollis [23, 24]. Thus, the clinical symptoms were directly related to the severe inflammatory reaction observed in the cerebral tissue of infected rabbits. This caused increased intracranial pressure, leading to the symptoms noted above [25]. Other potential causes of nerve pathology in rabbits include bacterial encephalitis, hydrocephalia, and malignant lymphoma [26]; however, none of these conditions were observed during necropsy or histopathological examination. The neurological symptoms were consistent with the presentation of the most severe CNS injuries (Grade III) and also with the presentation of the most severe nonsuppurative brain granulomas (Type C). However, some of the rabbits had discrete changes and showed no evidence of granulomas in the nervous tissue. This lack of association between the severity or degree of the brain lesions and the clinical behavior of the rabbits contrasts some previous findings, which suggested that the severity of the nervous tissue damage due to* E. cuniculi* infection is directly responsible for the manifestation of neurological symptoms [20, 26]. It is also important to note that the severity of the inflammatory reaction in the brain did not reflect the severity of the neurological symptoms [23]. In a retrospective study of 118 naturally infected rabbits, however, it was reported that the clinical manifestations such as torticollis and ataxia occurred more frequently in animals with the most severe brain injuries [22], and this is in agreement with the findings of our present study. Further, as noted elsewhere, gender was not a predisposing factor for the presentation of neurological symptoms [27].

This study is somewhat limited by the small number of animals studied and the fact that only rabbits with obvious neurological symptoms were examined. However, from our data, we infer that the relationship between host resistance and the infecting agent may exist in a balanced state [28]. We propose that extensive proliferation of* E. cuniculi* in the brain could lead to the rupture of the parasitic cysts, releasing infective spores directly into the adjacent nervous tissue [29]. This would cause the granulomatous reaction observed in most of the infected animals with Types B or C cerebral lesions, and this is consistent with the observation that spores and intact cysts were found adjacent to granulomatous reactions [30]. In support of this, it has been hypothesized that the chitin of endospore microsporidians is chemotactic for macrophages [31], leading to the recruitment of CD8^+^ cells and production of IL-2 and IFN-*γ* [32]. The ending result is a granuloma [1]. Few studies have attempted to classify and characterize* E. cuniculi* lesions in a natural infection [33]. Severe injuries have been reported in the cerebellum and meninges of rabbits naturally infected with* E. cuniculi* [34]; however, this did not occur in our study. Instead, we found that Grade III lesions, associated with Type C granulomas, were a common finding in the cerebral tissue. This also contrasts with other authors who found that the formation of granulomas was rare [20]. This difference could be attributed to the age of the animals, as the average age in most studies is 3 years old approximately, whereas in our study, the average age was 3 months (young adults). Our data strongly suggest that cerebral lesions are more severe in younger than in older individuals [35]. It is probable that severity of the lesions diminishes as the rabbits reach adulthood, because they are more resistant to* E. cuniculi* infection [5]. Although a classification of the size of the granulomas was not performed in that study [5], they reported granulomas that were smaller than those found in our study. According to the suggested grading system herein, the occurrence of smaller along with larger granulomas in the nervous tissue of older rabbits could indicate an* E. cuniculi* reinfection or recurrent parasitemia [33, 36].

In the kidney, most of the rabbits studied presented Grade III injuries, but unlike the severity of the lesions observed in the brain, the granulomas were rarely observed in the renal parenchyma. However, interstitial lymphocytic proliferation, necrosis of tubular epithelium, and interstitial fibrosis were more abundant in the kidneys and were directly associated with intact and ruptured cysts containing the parasite. Microscopically, the renal lesions were more severe than those observed in the cerebral tissue, suggesting that, in naturally infected young rabbits, the kidneys may be infected first [33, 37].

The most common lesions observed in infected livers were Grades II and III [38, 39]. It has been suggested that the severity of hepatic damage is influenced by the route of the parasite entry; that is, more severe lesions are observed when* E. cuniculi* spores are given orally [40]. This mode of transmission could have occurred for the animals in this study, since most of the livers examined displayed severe injuries. Some authors have attributed the hepatic lesions to a nonspecific immune response [36]. The perivascular distributions of the injury combined with the vasculitis were similar to those suggested with participation of immunopathological phenomena, like Types III and IV hypersensitivities, as it has been reported in the domestic dog (*Canis familiaris*) [41], and the squirrel monkey (*Saimiri sciureus*) [42]. The same immunopathological mechanism was suggested in blue foxes (*Alopex lagopus*) infected with* E. cuniculi* [43].

It is known that an individual's immunological status plays an important role in the elimination of* E. cuniculi* [23, 29, 44]. To date, only a few serological studies have been conducted worldwide to determine the prevalence of this pathogen in rabbits [45, 46]. Here, all rabbits sampled were seropositive for* E. cuniculi*. This is interesting, as positive rates for CIA testing in other studies ranged from 31 to 62% [18, 47, 48]. This test is used to screen for* E. cuniculi* in laboratory and pet rabbits in Europe, Asia, and USA [49], but it has not been used in our country and Latin America for that purpose. Although this study is the first to characterize and measure* E. cuniculi* infection in farm rabbits in the country, our data suggest for first time that* E. cuniculi* infection may be more common here than in other parts of the world [50]. Since this emerging pathogen is transmitted through feces and urine, the lack of rapid and specific diagnostic methods (e.g., the CIA test) in many regions could represent a serious zoonotic problem in the areas where these animals are bred, particularly for people involved either directly or indirectly in rabbit production [51]. One advantage of this test is that, compared with ELISA or IFAT, it provides comparable test results, and it is easy and quick to perform and does not require expensive equipment or specialized personnel [52]. Another advantage is that detection of* E. cuniculi*-specific IgG could identify latent infection, even in the absence of clinical symptoms. Similarly, the high frequency of spores observed in fecal and urine samples suggested an active infection. This study clearly demonstrated that this pathogen can be widespread in the facilities where rabbits are breeding and that these animals or others are capable of shedding the spores into the environment for an indefinite period of time. Here, only the fattening stage was studied; however, further studies will be required at other productions steps, including those assessing environmental reservoirs, such as the water source and food supply [53, 54]. This will be critical for identifying other possible routes of zoonotic transmission and establishing prevention and control measures.

Urine spores were observed in rabbits with the most severely injured renal tissue, coinciding with the reported observations by Pye and Cox [55], and confirm the observation of ruptured parasitic cysts and free spores within the lumen of renal tubules. This suggests a recurrent release of spores through the urine and contrasts with the findings of other authors, who reported an absence of spores in urine from rabbits with severe renal fibrosis and tissue repair [1, 56]. To date, the extent to which infective spores were disseminated via the urine of sick animals at the sampled farms remains unknown. More studies are needed to assess the health status of these farms with respect to parameters associated with animal welfare, such as environmental stress factors, management records, and physiological and productive indicators [57, 58]. The source of infection in the animals studied remains unknown, as this is the first time that* E. cuniculi* infection was observed at those farms. All animals came from the same rabbit supplier; therefore, it is likely that some animals were already infected when they arrived. We hypothesize that the stress of transportation could have triggered the active parasite infection and the subsequent dissemination of the disease to other rabbits in the facility. Further epidemiological studies, particularly those involving clinically healthy animals, will allow us to clarify the role of the rabbit in the geographical distribution of* E. cuniculi*.

In conclusion,* E. cuniculi* represents an important source of zoonosis, and this is the first confirmed report of this pathogen in farm meat rabbits in this part of the country. The main cerebral injuries were Grade III, coinciding with the presence of Type C granulomas. The cerebral lesions were located in the cortex, brain stem, and medulla. The renal lesions were also Grade III with no granulomas. The involvement of immunopathological phenomena (hypersensitivity Types III and IV) in Grade III lesions in kidney and liver is also suggested [59]. All rabbits were seropositive to* E. cuniculi*, indicating that this pathogen is widely distributed among animals intended for human consumption. In addition, we believe this work will provide direction and help to confirm the diagnosis of a suspected encephalitozoonosis. The grading results could be used as a guide when examining* E. cuniculi* lesions in tissues in Latin America veterinary laboratories.

## Figures and Tables

**Table 1:** Number of rabbits exhibiting the different grading in several organs/system.

Organ/system	Grade I	Grade II	Grade III
Central nervous system	30 (38%) (9 F-21 M)	16 (20%) (7 F-9 M)	35 (43.7%) (13 F-22 M)
Kidney	32 (40%) (23 F-9 M)	10 (13%) (2 F-8 M)	38 (48%) (11 F-27 M)
Liver	16 (20%) (5 F-11 M)	37 (46%) (13 F/24 M)	27 (34%) (17 F-10 M)

F: female; M: male.

**Table 2:** Number of rabbits showing the number of spores found in stools or urine.

Sample	Stain technique	Rare (about 1–5 spores)	Moderate (5–10 spores)	Many (more than 10 spores)
Stools	Trichrome blue	59 (74%) (25 F-34 M)	12 (15%) (7 F-5 M)	9 (11%) (3 F-9 M)
	Calcofluor	64 (80%) (25 F-34 M)	11 (14%) (9 F-8 M)	5 (6%) (3 F-9 M)

Urine	Trichrome blue	19 (24%) (6 F-13 M)	ND	ND
	Calcofluor	23 (29%) (11 F-12 M)	ND	ND

F: female; M: male.

ND: no data.

## References

[1] Harcourt-Brown F. M. (2004). *Encephalitozoon cuniculi* infection in rabbits. *Seminars in Avian and Exotic Pet Medicine*.

[2] Vavra J. L., Ronny Darson J. I., Weiss L. M., Becnel J. J. (2014). Structure of microsporidia. *Microsporidia: Pathogens of Opportunity*.

[3] Canning E. U., Hollister W. S. (1987). Microsporidia of mammals—widespread pathogens or opportunistic curiosities?. *Parasitology Today*.

[4] Künzel F., Joachim A. (2010). Encephalitozoonosis in rabbits. *Parasitology Research*.

[5] Mathis A., Weber R., Deplazes P. (2005). Zoonotic potential of the microsporidia. *Clinical Microbiology Reviews*.

[6] Al-Sadi H. I., Al-Mahmood S. S. (2014). Pathology of experimental *Encephalitozoon cuniculi* infection in immunocompetent and immunosuppressed mice in Iraq. *Pathology Research International*.

[7] Deplazes P., Mathis A., Baumgartner R. (1996). Immunologic and molecular characteristics of *Encephalitozoon*-like microsporidia isolated from humans and rabbits indicate that *Encephalitozoon cuniculi* is a zoonotic parasite. *Clinical Infectious Diseases*.

[8] Maddox J. V., Brooks W. M., Solter L. F., Navon A., Ascher K. R. S. (2011). Bioassays of microsporidia. *Bioassays of Entomopathogenic Microbes and Nematodes*.

[9] Baneux P. J. R., Pognan F. (2003). *In utero* transmission of *Encephalitozoon cuniculi* strain type I in rabbits. *Laboratory Animals*.

[10] Graczyk T. K., Sunderland D., Rule A. M. (2007). Urban feral pigeons (*Columba livia*) as a source for air- and waterborne contamination with *Enterocytozoon bieneusi* spores. *Applied and Environmental Microbiology*.

[11] Accoceberry I., Greiner P., Thellier M. (1977). Rabbit model for human intestinal microsporidia. *Journal of Eukaryotic Microbiology*.

[12] Rich G. (2010). Clinical update on testing modalities for *Encephalitozoon cuniculi* in clinically sick rabbits. *Journal of Exotic Pet Medicine*.

[13] Csokai J., Kunzel F. (2010). *Encephalitozoon-cuniculi*-Infektion bei Kaninchen. *Der Praktische Tierarzt*.

[14] Valencakova A., Revajova V., Balent P., Lešník F., Levkut M. (2003). Immunosuppressive effect of *Encephalitozoon cuniculi*. *Bulletin of the Veterinary Institute in Pulawy*.

[15] Anete Lallo M., Porta Miche Hirschfeld M. (2012). Encephalitozoonosis in pharmacologically immunosuppressed mice. *Experimental Parasitology*.

[16] van Gool T., Biderre C., Delbac F., Wentink-Bonnema E., Peek R., Vivarès C. P. (2004). Serodiagnostic studies in an immunocompetent individual infected with *Encephalitozoon cuniculi*. *Journal of Infectious Diseases*.

[17] Snowden K. F., Weiss L. M., Becnel J. J. (2014). Microsporidia in higher vertebrates. *Microsporidia: Pathogens of Opportunity*.

[18] Dipineto L., Rinaldi L., Santaniello A. (2008). Serological survey for antibodies to *Encephalitozoon cuniculi* in pet rabbits in Italy. *Zoonoses and Public Health*.

[19] Furuya K. (2009). Spore-forming microsporidian *Encephalitozoon*: current understanding of infection and prevention in Japan. *Japanese Journal of Infectious Diseases*.

[20] Csokai J., Gruber A., Künzel F., Tichy A., Joachim A. (2009). Encephalitozoonosis in pet rabbits (*Oryctolagus cuniculus*): pathohistological findings in animals with latent infection versus clinical manifestation. *Parasitology Research*.

[21] Chioralia G., Trammer T., Kampen H., Seitz H. M. (1998). Relevant criteria for detecting microsporidia in stool specimens. *Journal of Clinical Microbiology*.

[22] Künzel F., Gruber A., Tichy A. (2008). Clinical symptoms and diagnosis of encephalitozoonosis in pet rabbits. *Veterinary Parasitology*.

[23] Latney L. V., Bradley C. W., Wyre N. R. (2014). *Encephalitozoon cuniculi* in pet rabbits: diagnosis and optimal management. *Veterinary Medicine: Research and Reports*.

[24] Rebel-Bauder B., Leschnik M., Maderner A., Url A. (2011). Generalized encephalitozoonosis in a young kitten with cerebellar hypoplasia. *Journal of Comparative Pathology*.

[25] Percy D. H., Barthold S. W. (2007). *Pathology of Laboratory Rodents and Rabbits*.

[26] Gruber A., Pakozdy A., Weissenbock H., Csokai J., Künzel F. (2009). A retrospective study of neurological disease in 118 rabbits. *Journal of Comparative Pathology*.

[27] Shin J.-C., Kim D.-G., Kim S.-H., Kim S., Song K.-H. (2014). Seroprevalence of *Encephalitozoon cuniculi* in pet rabbits in Korea. *Korean Journal of Parasitology*.

[28] Didier E. S., Weiss L. M., Becnel J. J. (2014). Mammalian animal models of human microsporidiosis. *Microsporidia, Pathogens of Opportunity*.

[29] Valencakova A., Halanova M. (2012). Immune response to *Encephalitozoon* infection review. *Comparative Immunology, Microbiology & Infectious Diseases*.

[30] Khan I. A., Moretto M., Weiss L. M. (2001). Immune response to *Encephalitozoon cuniculi* infection. *Microbes and Infection*.

[31] Rodriguez-Tovar L. E., Speare D. J., Markham R. J. F. (2011). Fish microsporidia: immune response, immunomodulation and vaccination. *Fish and Shellfish Immunology*.

[32] Ghosh K., Schwartz D., Weiss L. M., Weiss L. M., Becnel J. J. (2014). Laboratory diagnosis of microsporidia. *Microsporidia, Pathogens of Opportunity*.

[33] Leipig M., Matiasek K., Rinder H. (2013). Value of histopathology, immunohistochemistry, and real-time polymerase chain reaction in the confirmatory diagnosis of *Encephalitozoon cuniculi* infection in rabbits. *Journal of Veterinary Diagnostic Investigation*.

[34] Eröksüz H., Eröksüz Y., Metin N., Özer H. (1999). Morphologic examinations of cases of naturally acquired encephalitozoonosis in a rabbit colony. *Turkish Journal of Veterinary and Animal Sciences*.

[35] Furuya K., Sugiyama H., Ohta M., Nakamura S., Une Y., Sasaki S. (2011). Cerebral microsporidiosis caused by *Encephalitozoon cuniculi* infection in a young squirrel monkey. *Journal of Neuroparasitology*.

[36] Mathews A., Hotard A., Hale-Donze H. (2009). Innate immune responses to *Encephalitozoon* species infections. *Microbes and Infection*.

[37] Jass A., Matiasek K., Henke J., Küchenhoff H., Hartmann K., Fischer A. (2008). Analysis of cerebrospinal fluid in healthy rabbits and rabbits with clinically suspected encephalitozoonosis. *Veterinary Record*.

[38] Botha W. S., Stewart C. G., van Dellen A. F. (1986). Observations on the pathology of experimental encephalitozoonosis in dogs. *Journal of the South African Veterinary Association*.

[39] Wicher V., Baughn R. E., Fuentealba C., Shadduck J. A., Abbruscato F., Wicher K. (1991). Enteric infection with an obligate intracellular parasite, *Encephalitozoon cuniculi*, in an experimental model. *Infection and Immunity*.

[40] Fuentealba I. C., Mahoney N. T., Shadduck J. A., Harvill J., Wicher V., Wicher K. (1992). Hepatic lesions in rabbits infected with *Encephalitozoon cuniculi* administered per rectum. *Veterinary Pathology*.

[41] Szabo J. R., Shadduck J. A. (1987). Experimental encephalitozoonosis in neonatal dogs. *Veterinary Pathology*.

[42] Zeman D. H., Baskin G. B. (1985). Encephalitozoonosis in squirrel monkeys (*Saimiri sciureus*). *Veterinary Pathology*.

[43] Åkerstedt J. (2003). Humoral immune response in adult blue foxes (*Alopex lagopus*) after oral infection with *Encephalitozoon cuniculi* spores. *Veterinary Parasitology*.

[44] Didier E. S., Khan I. A., Weiss L. M., Becnel J. J. (2014). The immunology of microsporidiosis in mammals. *Microsporidia*.

[45] Boot R., Hansen A. K., Hansen C. K., Nozari N., Thuis H. C. W. (2000). Comparison of assays for antibodies to *Encephalitozoon cuniculi* in rabbits. *Laboratory Animals*.

[46] Didier E. S. (2000). Immunology of microsporidiosis. *Contributions to Microbiology*.

[47] Didier E. S. (1998). Microsporidiosis. *Clinical Infectious Diseases*.

[48] Santaniello A., Dipineto L., Rinaldi L. Sieroprevalenza di *Encephalitozoon cuniculi* in allevamenti intensivi di conigli.

[49] Greenstein G., Drozdowicz C. K., Garcia F. G., Lewis L. L. (1991). The incidence of *Encephalitozoon cuniculi* in a commercial barrier-maintained rabbit breeding colony. *Laboratory Animals*.

[50] Keeble E. J., Shaw D. J. (2006). Seroprevalence of antibodies to *Encephalitozoon cuniculi* in domestic rabbits in the United Kingdom. *Veterinary Record*.

[51] Bornay-Llinares F. J., Da Silva A. J., Moura H. (1998). Immunologic, microscopic, and molecular evidence of *Encephalitozoon intestinalis* (*Septata intestinalis*) infection in mammals other than humans. *Journal of Infectious Diseases*.

[52] Sak B., Kučerová Z., Kváč M., Květoňová D., Rost M., Secor E. W. (2010). Seropositivity for *Enterocytozoon bieneusi*, Czech Republic. *Emerging Infectious Diseases*.

[53] Bigliardi E., Sacchi L. (2001). Cell biology and invasion of the microsporidia. *Microbes and Infection*.

[54] Cerioli M., Brivio R., Grilli G. Search for the key health and welfare indicators for meat rabbit production and definition of a score method of evaluation.

[55] Pye D., Cox J. C. (1977). Isolation of *Encephalitozoon cuniculi* from urine samples. *Laboratory Animals*.

[56] Furuya K., Asakura T., Igarashi M., Morita T. (2009). Microsporidian *Encephalitozoon cuniculi* antibodies in rabbit urine samples. *Veterinary Record*.

[57] Sak B., Kváč M., Kučerová Z., Květoňová D., Saková K. (2011). Latent microsporidial infection in immunocompetent individuals—a longitudinal study. *PLoS Neglected Tropical Diseases*.

[58] Fukui D., Bando G., Furuya K. (2013). Surveillance for an outbreak of *Encephalitozoon cuniculi* infection in rabbits housed at a zoo and biosecurity countermeasures. *Journal of Veterinary Medical Science*.

[59] Ghosh K., Weiss L. M. (2012). T cell response and persistence of the microsporidia. *FEMS Microbiology Reviews*.

